# Synthesis of the Tetragonal Phase of Zintl’s NaTl and Its Structure Determination from Powder Diffraction Data

**DOI:** 10.3390/ma12081356

**Published:** 2019-04-25

**Authors:** Susanne Tiefenthaler, Nikolaus Korber, Stefanie Gärtner

**Affiliations:** 1 Institute of Inorganic Chemistry, University of Regensburg, 93040 Regensburg, Germany; Susanne.Tiefenthaler@ur.de (S.T.); Nikolaus.Korber@ur.de (N.K.); 2 Central Analytics (X-Ray Dept.), University of Regensburg, 93040 Regensburg, Germany

**Keywords:** Zintl phase, NaTl, ammonia, X-ray powder diffraction

## Abstract

A tetragonal distortion of the long-time known NaTl structure at 298 K was observed in different experimental setups, including Zintl’s original procedure of reducing Tl(I)-iodide by sodium liquid ammonia solutions. The powder diffraction pattern obtained by the high temperature synthesis using classical solid-state techniques allowed a model-independent unambiguous structure solution and refinement of tetragonal distorted NaTl (Rp = 0.0179, wRp = 0.0246, R = 0.0477, wR = 0.0527, GooF = 1.24).

## 1. Introduction

The synthesis of intermetallic compounds predominantly is carried out at high temperatures using well-known solid-state reaction techniques, but for one special class of compounds a low temperature route is possible, if alkali elements are involved, as they are soluble in anhydrous liquid ammonia under formation of blue colored alkalimetal ammonia solutions [1]. The blue color arises from free electrons, which are able to reduce more electronegative main group elements under formation of negatively charged main group ions. For Groups 14 (Sn and Pb) and 15 (P–Bi), the resulting molecular polyanions are stable in these solutions, whereas Group 13 elements react under formation of insoluble fine powdery materials [2,3]. The first observations concerning this low temperature route go back to the end of the nineteenth century, when Joannis had first observed a reaction between lead and sodium in liquid ammonia [4], which was subsequently investigated more in-depth by Kraus [5]. More than three decades later, Eduard Zintl started his investigations of the synthesis of polar intermetallic compounds in liquid ammonia, by reducing metal-halides using dissolved sodium metal [6]. Besides a large number of Group 14 and Group 15 polar intermetallics following the (8−N) rule — which are now also described in terms of the Zintl–Klemm concept [7,8,9] — he also was able to determine the crystal structure of microcrystalline samples of NaTl, synthesized via the reduction of thallium(I)iodide by sodium in liquid ammonia (equation (1)) using powder diffraction methods [10]. The preparation of powder samples for X-ray diffraction experiments under inert conditions at times before glove boxes was not trivial, and Zintl et al. reported, in 1931, on an apparatus for sample preparation for air and moisture sensitive alloys [11]. According to Zintl, his experimental setup caused broad Debye lines due to lattice defects. Nevertheless, he rightly concluded from his observed data that both thallium and sodium crystallize in interwoven diamond sublattices, forming a face-centered cubic structure (space group *Fd*-3*m*) [10]. The determination of the sodium thallide structure represents a milestone in intermetallic chemistry as it proves the applicability of a formal electron transfer from the electropositive element to the electronegative under formation of salt-like structures following the pseudo element concept for the resulting polyanion [7]. Therefore, the cubic sodium thallide structure represents a fundamental structure type (B32), which is subject of undergraduate and graduate lectures in inorganic solid-state chemistry and is present in all corresponding textbooks. A number of binary and ternary phases have since been found to crystallize in the NaTl structure type, having been named after the initial compound. In addition to the sodium compound, Zintl also determined the structure of the binary thallide with lithium in the composition 1:1. Due to the cation size however, this compound crystallizes in the β-brass structure type, rather than the NaTl one [12].

Despite being an interesting field with a lot of potential for further explorations in terms of fundamental research, due, in part, to its toxicity and lack of applications, thallium compounds have since largely faded from the focus of inorganic chemistry.

While it has been known for a century, that the compound KTl exists [13], but does not crystallize in the NaTl structure [14], only in 1993, Corbett et al. reported on the crystal structure of the second binary alkali metal thallide KTl [15]. In contrast to the compound containing the lighter homologue sodium, thallium does not form a diamond lattice, rather isolated Tl_6_^6−^ ions [15]. These strongly compressed octahedra are also present in the analogous structure of CsTl [16]. So far, at ambient conditions, no binary compound of rubidium and thallium with a composition of 1:1 is known.

Working with the methods available at the time, Zintl accomplished great things in solving the structure from powder data. However, since the 1930s, experimental standards and the characterization methods available have greatly improved, allowing a more in-depth view into the long known structures

The phase transitions of A^I^B^III^ compounds have been extensively studied in the context of the formation of β-brass type structures at high pressures [17,18]. Investigations on NaTl itself are very rare; in the 1970s melting entropies were determined without any evidence for a phase transition similar to LiIn [19]. In 1991 neutron scattering experiments up to 900 K yielded a mobility of the sodium atoms in NaTl, but a tetragonal distortion was not observed [20]. Altogether there are ten phases which crystallize in the NaTl structure type (LiAl [21], LiGa, LiIn, LiZn, LiCd [12], NaIn [22], NaTl [10], hp-KTl [17], hp-RbTl, and hp-CsTl [18]). Hp-KTl [17], hp-RbTl, and hp-CsTl are only stable at high pressure (hp) [18], two of the remaining seven (LiIn and LiAl) show a tetragonal distortion derived by a phase transition between translationengleiche space groups from cubic *Fd*-3*m* to *I*4_1_/*amd* [23,24]. For LiAl a transition temperature of 93 K was determined, for LiIn the transition occurs at a temperature of 170 K. Both structures show a similar degree of distortion η (= c/($\sqrt{2}$a)), however the former is closer to the cubic structure with η = 1.0017, while LiIn is slightly more distorted with η = 1.0047.

The repetition of Zintl’s original experiments in the context of a student’s research lab course produced unexpected results in the form of a powder diffraction pattern originating from a tetragonal unit cell rather than a cubic one. In order to verify these findings, we performed more experiments, producing NaTl in a number of ways. Here we report on our investigations of Zintl’s NaTl using his preparation method in liquid ammonia as well as high temperature synthesis of NaTl, which allowed an unequivocal determination of a tetragonal distortion of NaTl at ambient conditions.

## 2. Materials and Methods

All steps were performed under argon atmosphere, either in a glovebox or through application of Schlenk techniques. Since ammonia is gaseous at room temperature (boiling point 240 K), all reaction vessels were cooled with an ethanol/CO_2_ bath during the preparation of the NH_3_ solutions. The reactions vessels were stored at 238 K in the freezer to allow for reaction. Due to the high sensitivity towards water or oxygen of the compounds, all products were handled and stored in an argon filled glovebox Labmaster130 (MBraun, Garching, Germany).

All samples were prepared from the following reactants; sodium (98%, Merck Darmstadt, Darmstadt, Germany segregated before use), thallium (99.999%, Strem Chemicals, Newburyport, MA, USA), TlI (99.999%, ABCR, Karlsruhe, Germany), TlBr (99.999%, Alfa Aesar, Haverhill, MA, USA), TlPF_6_ (97%, Strem Chemicals, Newburyport, MA, USA), and TlBF_4_ (prepared from Tl, HBF_4_, and H_2_O_2_).

### 2.1. NaTl Via Direct Reduction in Liquid Ammonia

In accordance with Zintl’s original experiments, sodium and thallium (I) salts were reacted in liquid ammonia. Sodium (11.3 mg, 0.49 mmol) and thallium(I)bromide (70 mg, 0.25 mmol) were placed into Schlenk tubes (dried in vacuo before use) and subsequent condensation of dry liquid ammonia resulted in the formation of a dark blue solution caused by the presence of solvated electrons. The desired reaction followed equation 1. In addition to Zintl’s original procedure of reducing thallium(I) iodide by elemental sodium, we also used different thallium(I) salts (TlBr, TlBF_4_, and TlPF_6_), to ascertain that our findings were not caused by unknown effects produced by the counterion. For all of these experiments, 18.4 mg of sodium (0.8 mmol) were used, as we determined this amount to be ideal for the characterization of the product. The exact amounts of the used thallium(I) salts are listed in Table 1. After several days of storage at 238 K, the blue color of the solutions had faded, indicating the completeness of the reaction, leaving a colorless liquid and a macroscopically gray precipitate consisting of black and white crystalline material. Following the removal of the ammonia, the fine powder was dried in vacuo and transferred into a glove box for further storage.

2Na + TlX → NaTl + NaX (X = I, Br, PF_6_, BF_4_) (1)

**Figure 1:**
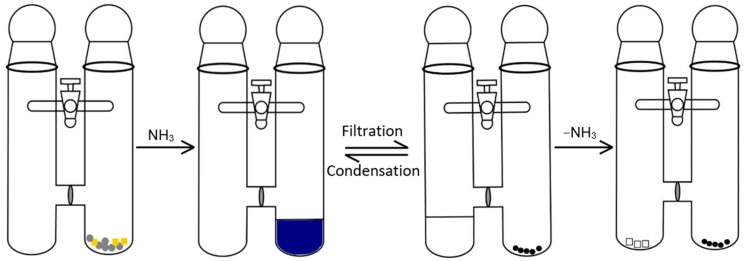
Schematic drawing of the experimental process. Yellow squares represent thallium(I) salts, gray circles represent sodium, white squares represent sodium(I) salts, and black circles represent NaTl. Once the solution had turned colorless, the ammonia was poured through the frit into the second leg and condensed back onto the reactants, this procedure was repeated three times.

In addition to the experiments performed in Schlenk tubes, the tests were repeated in reaction vessels, where two legs were separated by a semiporous frit (“H-tubes”) in order to wash out the sodium halide and other soluble byproducts. For this purpose, the reactants were weighed into one leg of the flask followed by condensation of approximately 10 mL of dry liquid ammonia only on the reactant side. For these experiments, double the amounts of reactants were used (1.6 mmol sodium, 0.8 mmol TlX), as there is some wastage due to the filtration process. Once the solution had turned colorless, the liquid was poured through the frit into the other leg, separating the precipitation from the halide solution. Subsequent condensation of the ammonia from the one leg back onto the reactant side allowed for the repetition of the process until all soluble byproducts were extracted from the reaction products. A schematic drawing for the process is shown in Figure 1.

### 2.2. NaTl Via High Temperature Reaction

**Scheme 1:**
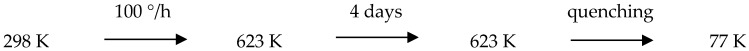
Temperature profile for the high temperature synthesis of NaTl.

In order to verify the findings without the possibility of an effect of the byproducts on the structure, and as the products obtained via the original Zintl route are impure, in additional experiments NaTl was synthesized via high temperature reaction. Sodium (115.3 mg, 5.02 mmol) and thallium (1025.6 mg, 5.02 mmol) were placed into a fused silica ampoule in a glove box (argon atmosphere). The ampoule was sealed by melting and was allowed to react according to Scheme 1. Subsequently, the ampoule was quenched in liquid nitrogen. The received brittle solid was ground up in a mortar under argon atmosphere, delivering a shiny metallic powder.

### 2.3. Characterization of NaTl

For powder X-Ray diffraction analysis of the samples, glass capillaries with a diameter of 0.3 mm were filled with the reaction products under argon atmosphere (glovebox) and sealed by melting. All samples were measured on a diffractometer STADI P (STOE & Cie GmbH, Darmstadt, Germany), equipped with a molybdenum sealed tube micro focus radiation source at room temperature. The samples prepared according to the original Zintl route were measured in the 2Θ range between 8° and 37°, while the sample synthesized via high-temperature reaction was measured in the 2Θ range between 2° and 47°. For visualization and indexing purposes, the software WinXPOW (STOE & Cie GmbH, Darmstadt, Germany) was used [25]. All crystallographic data used for reference was calculated from CIF (crystallographic information file) data deposited in the ICSD (Inorganic Crystal Structure Database). For the determination of the crystal structure from the diffraction pattern JANA2006 [26] software (Freeware supported by the Czech Science Foundation and Academy of Sciences of the Czech Republic, Institute of Physics) was used. ǀF_hkl_ǀ values were extracted from the rebuilt X-ray powder pattern by applying the LeBail method by taking the peak asymmetry by the fundamental parameter approach into account [27]. For structure solution, charge-flipping methods have been applied (Superflip [28]). The refinement of the structure was performed by Rietveld methods [29], implemented in JANA2006.

## 3. Results and Discussion

A quick first glance at the observed powder diffraction patterns confirmed the presence of NaTl in all samples. However, by taking a closer look at the observed diffractograms a splitting of some of the reflections compared to Zintl’s NaTl became evident. The diffractogram of the repetition of Zintl’s original experiment is shown in the (SM Figure S1) and the published data of cubic NaTl (*Fd*-3*m*) is given for comparison [10]. As expected, reflections associated with NaI are observable, as well as reflections characteristic for elemental thallium. As residual elemental thallium also means an excess of elemental sodium we assume the formation of sodium amide as the product of a well-known side reaction—2 Na + 2 NH_3_ → 2 NaNH_2_ + H_2_—in small quantities, not observable in the powder diffraction patterns (<5%).

Extraction of the sodium salts lead to cleaner powder data, hence allowing for a better interpretation of the data. The collected powder data from a washed batch of NaTl is depicted in the (SM Figure S2). The collected data clearly shows splitting of some reflections, which are characteristic for NaTl. A careful analyses of observable reflections confirmed the presence of a tetragonal body centered structure (tI: h + k + l = 2n) in contrast to the cubic face centered (cF: h, k, l all even or all odd) structure reported so far. Traces of impurities did not allow for an unequivocal identification of the reflection pattern. Since both NaTl and elemental thallium produce a strong reflection at 2Θ = 15.47°, further experiments had to be performed, in order to be certain of the structure.

The low temperature preparation method in liquid ammonia of course does not allow a statement about the stoichiometry, as additional thallium is also observed and the side reaction of sodium with ammonia also takes place in a small amount. Therefore, we prepared phase pure, stoichiometric samples in fused silica ampoules which did not show any evidence for nonreacted thallium or sodium. Previous reported studies on the LiIn phase [23] showed a dependence of Li deficiency on the degree of the tetragonal distortion (the phase deficient in Li showed a smaller degree of distortion than the stoichiometric phase), but the phase transition also takes place for the stoichiometric compound.

**Figure 2:**
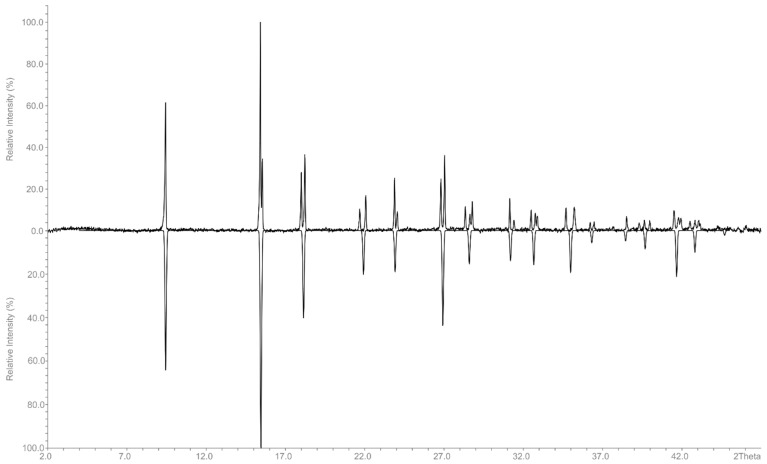
Powder X-ray diffraction pattern of NaTl produced by melting the elements and quenching the reaction (Mo radiation (λ = 0.70926 Å (Mo-K_α1_)) yields a smaller 2θ range compared to Cu radiation (λ = 1.540598 Å (Cu-K_α1_)), which is typically used). Downwards, Zintl’s cubic NaTl is given for comparison.

All observed reflections in the phase pure material obtained by the solid-state route ((Figure 2, upwards); for the synthesis see Section 2.2) are also present in the diffractograms of the powders produced by Zintl’s original low temperature route in liquid ammonia (see Supplementary Materials Figure S1).

**Figure 3:**
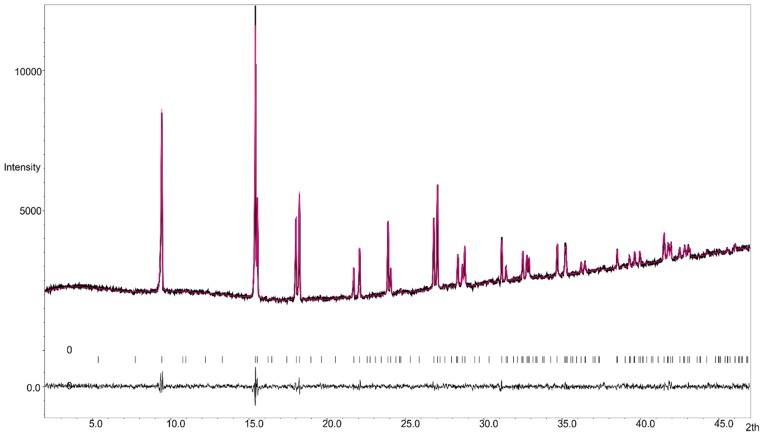
Fitted powder profile. Observed (black), fitted (pink), and difference profiles of tetragonal NaTl. (Mo radiation (λ = 0.70926 Å (Mo-K_α1_)) yields a smaller 2θ range compared to Cu radiation (λ = 1.540598 Å (Cu-K_α1_)), which is typically used.)

As no impurities are present, the collected data allowed further examination. Pattern fitting and subsequent structure solution and refinement (see Materials and Methods 2.3) allowed for an unequivocal structure determination by powder diffraction methods of tetragonal NaTl. The space group *I*4_1_/*amd* was determined by analyses of the observed reflections and subsequent structure solution by charge-flipping methods resulted in one Tl position. Following the Rietveld refinement, the inspection of Fourier difference maps revealed the presence of one maximum not in the area of thallium, which consecutively was refined as a sodium atom. Figure 3 shows the final pattern fit for tetragonal NaTl, in Table 2 crystallographic data is given, further information can be obtained from the CCDC (Cambridge Crystallographic Data Centre) or ICSD (Inorganic Crystal Structure Database) by quoting CSD 1898868.

Atom positions, isotropic displacement parameter (B_iso_), and crystallographic sites are given in Table 3; anisotropic displacement parameters (ADP’s) are given in Table 4. The comparably large ADP of sodium we assume is due to its thermal motion, as the free refinement of this site resulted in a value of 0.89(6) and no significantly smaller ADP’s. Therefore, the value was fixed at 1.0 (according to site symmetry of 0.125).

**Figure 4:**
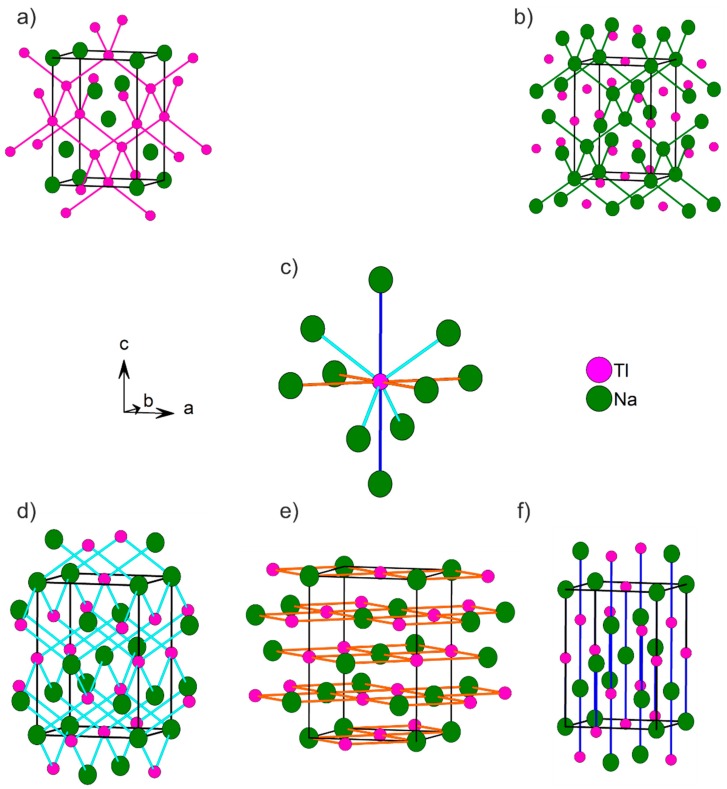
Distances in NaTl: (**a**) Diamond sublattice of thallium in NaTl; (**b**) sodium sublattice in NaTl; (**c**) coordination of thallium in NaTl with the different Tl–Na distances depicted in different colors (d(Tl–Na) dark blue = 3.7645(3) Å, d(Tl–Na) light blue = 3.22363(17) Å, d(Tl–Na) orange = 3.70105(19) Å); (**d**) the short Tl–Na distance results in a tetrahedral network with thallium and sodium being involved in the unit cell; (**e**) compressed Tl–Na distance is situated in the a/b plane of the unit cell; and (**f**) the elongated Tl–Na distance is located parallel to the c-axis in the unit cell.

The tetragonal structure of NaTl is isotypic to the low temperature structures of LiIn, respectively, LiAl. The asymmetric unit consists of two atoms (Table 2) located on special sites which only gives one Tl–Tl, respectively, Na–Na distance (3.22363(17) Å) in accordance with the cubic structure. In the cubic description there exist two Na–Tl distances (3.231 Å and 3.731 Å), in the discussed tetragonal compound the longer distance splits into 3.70105(19) Å and 3.7645(3) Å, with an average value very close to the cubic description. As can be seen in Table 5, in both the cubic and the tetragonal NaTl structures, each sodium and thallium atom, respectively, is coordianted tetrahedrally by four atoms of the same sort, leading to a diamond sublattice. These lattices are depicted in Figure 4a (thallium sublattice, pink in Figure 4) and Figure 4b (sodium sublattice, green in Figure 4). In addition to the tetrahedron comprised of the homoatomic atoms, each sodium atom is tetrahedrally coordinated by four thallium atoms and vice versa. This coordination is shown in Figure 4c,d with turquoise lines. The distances Na–Na, Tl–Tl, and Na–Tl are all equal. As a result of the tetragonal distortion the homoatomic, as well as the heteroatomic distances of the first shell are compressed, from 3.231 Å in the cubic system to 3.22363(17) Å in the tetragonal cell. While the coordination of each sodium and thallium atom respectively in the cubic system amounts to a regular octahedron with a Na–Tl distance of 3.731 Å, the tetragonal distortion leads to an elongation of the Na–Tl distance along the c-axis. In contrast, in the a–b plane, the Na–Tl distances are shortened (3.70105(19) Å) (Figure 4c, orange lines, Figure 4e). Hence, the octahedral environment is no longer regular, but slightly distorted. The different distances are shown in different colors in Figure 4c. Blue lines represent the longest Na–Tl distance (3.7645(3) Å), running parallel to the c-axis (Figure 4f). Additionally, as a consequence of the tetragonal distortion, two angles for the distorted tetrahedral environment of thallium, respectively, sodium in their diamond sublattices are observed (108.550(3)° and 109.9336(13)°), instead of the ideal tetrahedral angle (across (−1/3) = 109.471°) in the cubic case. The tetragonal distortion in NaTl is η = $c/\sqrt{2a}$ = 1.017, hence the distortion is more pronounced as in LiIn (η = 1.0045) and LiAl (η = 1.0017) [23,24].

## 4. Conclusions

The low temperature route for NaTl according to Zintl’s original procedure, after washing out the soluble side products, allows the isolation of a pure NaTl powder with a tetragonally distorted structure compared to the previously reported cubic NaTl phase. The high temperature route also results in the formation of tetragonally distorted NaTl, of which the structure was unambiguously determined by structure determination from powder diffraction data by charge flipping and Rietveld methods. This finding is supported by the previously reported results for LiIn and LiAl, which both undergo a phase transition between translationengleiche space groups from cubic *Fd*-3*m* to tetragonal *I*4_1_/*amd* upon cooling [23,24]. A similar phase transition for NaTl can be expected, deeper investigations are a clear demand and are currently in progress. The comparably high temperature regime for the stability of the tetragonal phase also allows further cooling which also needs to beinvestigated more in detail, as an even stronger distortion towards the orthorhombic crystal system cannot be excluded. The reasons for the NaTl structure being tetragonal instead of cubic at ambient conditions might be found in a different population of the thallium 6p states, which give the most important contribution to the bonding between the Tl atoms. However, the innocence of the sodium atoms concerning the distortion cannot yet be verified.

## Figures and Tables

**Table 1:** Amounts of the used thallium(I) salts.

Thallium(I) Salt	m(TlX) [mg]	n(TlX) [mmol]
TlI	132.5	0.08
TlBF_4_	116.5	0.08
TlPF_6_	139.7	0.08

**Table 2:** Crystallographic data for tetragonal sodium thallide.

Compound	NaTl
Formula weight/gmol^−1^	227.36
Color	gray
Crystal system	tetragonal
Space group	*I*4_1_/*amd*
a/Å	5.23408(19)
c/Å	7.5290(3)
Cell volume/Å^3^, Z	206.261(13), 4
Diffractometer	STOE STADI P Mythen 1K Mo-K_α1_ λ = 0.70926 Å
Absorption coefficient µ(Mo-Kα_1_)/mm^−1^	784.5
Temperature/K	298
2θ range/°	2.000 ≤ 2Θ ≤ 46.985
No. of measured points	3000
No. of parameters, constraints	10/0
Program	JANA2006 [Lit]
Profile function	Pseudo-Voigt
Background function	Manual background
Final Rp, wRp	0.0179, 0.0246
Final R/wR (obs. Reflections)	0.0480, 0.0527
Final R/wR (all reflections)	0.0477, 0.0527
GooF	1.24

**Table 3:** Atom positions, crystallographic sites, and Biso [Å^2^] of the atoms in tetragonal sodium thallide.

Atom	Site	x/a	y/b	z/c	Biso [Å^2^]
Tl	4*b*	0.5	0.5	0	0.0405(5)
Na	4*a*	0	0.5	0.25	0.160(14)

**Table 4:** Anisotropic displacement in Å^2^ parameters of the atoms in tetragonal sodium thallide.

Atom	U11	U22	U33	U23	U13	U12
Tl	0.0494(9)	0.0494(9)	0.0227(11)	0	0	0
Na	0.145(17)	0.145(17)	0.19(3)	0	0	0

**Table 5:** Distances in the cubic and tetragonal systems. Tl–Tl distances are equal to the Na–Na distances and therefore not explicitly mentioned.

Cubic	Tetragonal					
Atoms	Distance [Å]	Number	Atoms	Distance [Å]	Number	Color of Bond in Figure 4
Na–Na	3.231	4	Na–Na	3.22363(17)	4	pink
Na–Tl	3.231	4	Na–Tl	3.22363(17)	4	turquoise
Na–Tl	3.731	6	Na–Tl	3.70105(19)	4	orange
			Na–Tl	3.7645(3)	2	blue
Na–Na	5.276	12	Na–Na	5.234	4	not depicted
			Na–Na	5.279	8	not depicted

## Supplementary Materials

The following are available online at https://www.mdpi.com/1996-1944/12/8/1356/s1, Figure S1: Powder X-ray diffraction pattern of the powder received from the reaction 2 Na + TlI in liquid ammonia. Our observed diffraction pattern is depicted in black in the upper panel of the figure, while the calculated structure of Zintl’s product is shown in the downward facing peaks. Red lines indicate reflections belonging to NaI, while blue lines show the position of the reflections of elemental thallium, Figure S2: Powder X-ray diffraction pattern of washed NaTl. The measured data is shown with positive intensities, whereas the calculated data is depicted with negative intensities. The reflection at 2Θ = 14.8° is evidence of traces of elemental thallium still in the sample.
