# Supplementary material for: Synthesis of the Tetragonal Phase of Zintl’s NaTl and Its Structure Determination from Powder Diffraction Data
Source: Materials (Basel). 2019 Apr 25;12(8):1356. doi: 10.3390/ma12081356 (PMC6515420; doi:10.3390/ma12081356)
Supplement: Supplementary file 1 [file materials-12-01356-s001.pdf]

materials

Supplementary Materials

Synthesis of the Tetragonal Phase of Zintl's NaTl and its Structure Determination from Powder Diffraction Data

Susanne Tiefenthaler [1], Nikolaus Korber [1] and Stefanie Gärtner [1,2,*]

[1] Institute of Inorganic Chemistry, University of Regensburg, 93040 Regensburg, Germany; Susanne.Tiefenthaler@ur.de (S.T.); Nikolaus.Korber@ur.de (N.K.)
[2] Central Analytics (X-Ray Dept.), University of Regensburg, 93040 Regensburg, Germany
* Correspondence: Stefanie.Gaertner@ur.de; Tel.: +49-941-943-4446

Received: 14 March 2019; Accepted: 11 April 2019; Published: 25 April 2019

Powder diffraction data of NaTl produced via the original Zintl experiment (Fig. S1) and a washed batch of NaTl produced in an H-tube (Fig. S2).

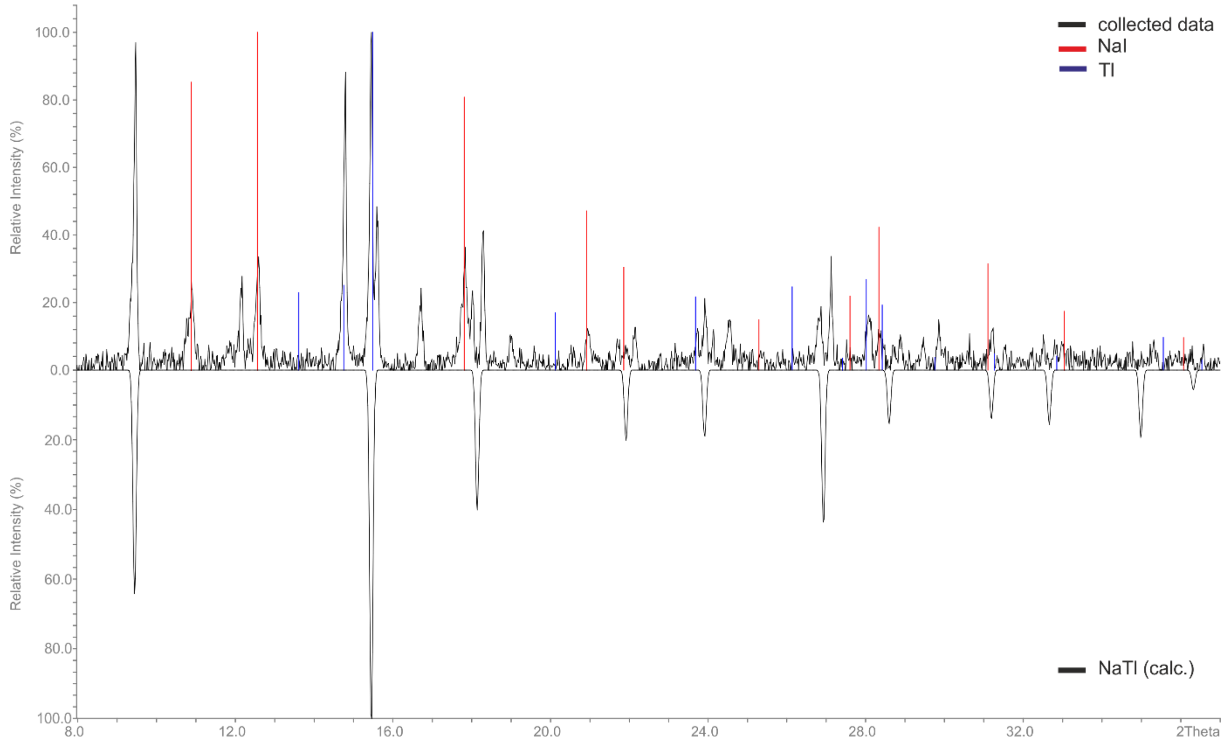

Figure S1. Powder X-ray diffraction pattern of the powder received from the reaction 2 Na + TlI in liquid ammonia. Our observed diffraction pattern is depicted in black in the upper panel of the figure, while the calculated structure of Zintl's product is shown in the downward facing peaks. Red lines indicate reflections belonging to NaI, while blue lines show the position of the reflections of elemental thallium.

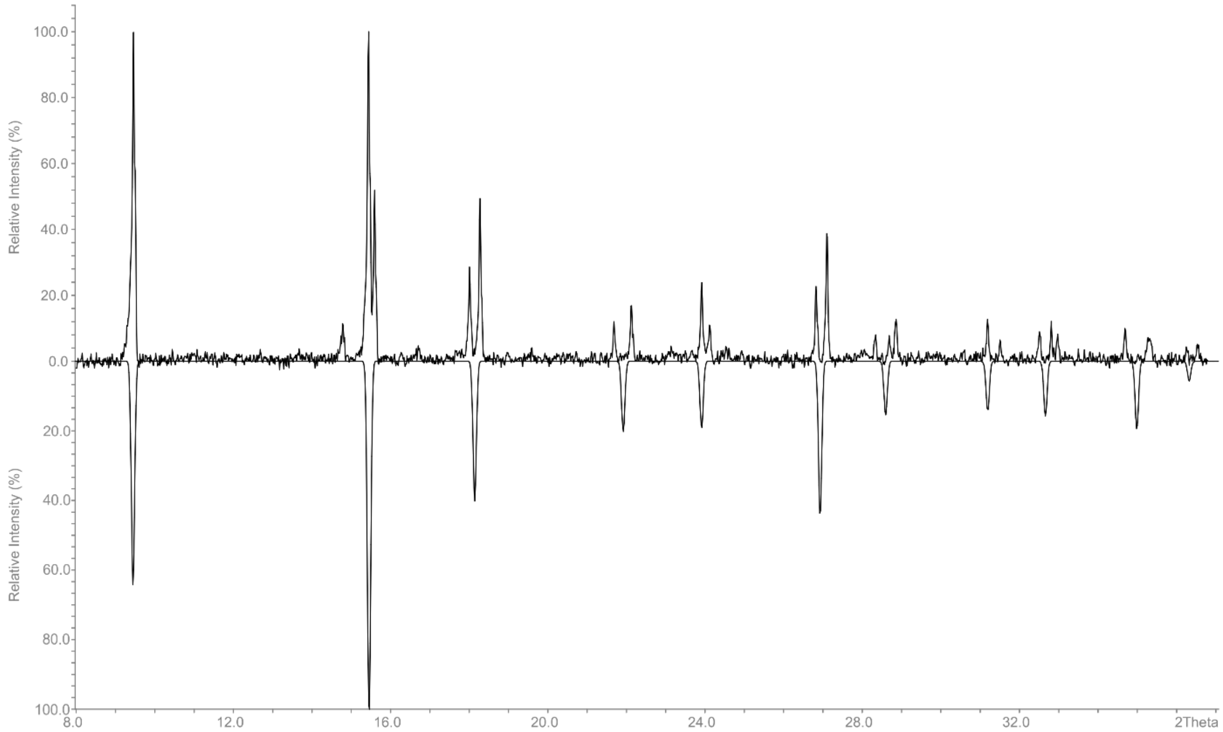

Figure S2. Powder X-ray diffraction pattern of washed NaTl. The measured data is shown with positive intensities, whereas the calculated data is depicted with negative intensities. The reflection at $2\Theta = 14.8°$ is evidence of traces of elemental thallium still in the sample.
